# Predicting the risk of nodular thyroid disease in coal miners based on different machine learning models

**DOI:** 10.3389/fmed.2022.1037944

**Published:** 2022-11-25

**Authors:** Feng Zhao, Hongzhen Zhang, Danqing Cheng, Wenping Wang, Yongtian Li, Yisong Wang, Dekun Lu, Chunhui Dong, Dingfei Ren, Lixin Yang

**Affiliations:** ^1^The First Hospital of Anhui University of Science & Technology (Huainan First People’s Hospital), Huainan, China; ^2^Anhui University of Science and Technology College of Medicine, Huainan, China; ^3^Graduate School of Bengbu Medical College, Bengbu, China; ^4^Occupational Control Hospital of Huai He Energy Group, Huainan, Anhui, China

**Keywords:** underground environment, coal miners, nodular thyroid disease, machine learning, predictive models

## Abstract

**Background:**

Nodular thyroid disease is by far the most common thyroid disease and is closely associated with the development of thyroid cancer. Coal miners with chronic coal dust exposure are at higher risk of developing nodular thyroid disease. There are few studies that use machine learning models to predict the occurrence of nodular thyroid disease in coal miners. The aim of this study was to predict the high risk of nodular thyroid disease in coal miners based on five different Machine learning (ML) models.

**Methods:**

This is a retrospective clinical study in which 1,708 coal miners who were examined at the Huaihe Energy Occupational Disease Control Hospital in Anhui Province in April 2021 were selected and their clinical physical examination data, including general information, laboratory tests and imaging findings, were collected. A synthetic minority oversampling technique (SMOTE) was used for sample balancing, and the data set was randomly split into a training and Test dataset in a ratio of 8:2. Lasso regression and correlation heat map were used to screen the predictors of the models, and five ML models, including Extreme Gradient Augmentation (XGBoost), Logistic Classification (LR), Gaussian Parsimonious Bayesian Classification (GNB), Neural Network Classification (MLP), and Complementary Parsimonious Bayesian Classification (CNB) for their predictive efficacy, and the model with the highest AUC was selected as the optimal model for predicting the occurrence of nodular thyroid disease in coal miners.

**Result:**

Lasso regression analysis showed Age, H-DLC, HCT, MCH, PLT, and GGT as predictor variables for the ML models; in addition, heat maps showed no significant correlation between the six variables. In the prediction of nodular thyroid disease, the AUC results of the five ML models, XGBoost (0.892), LR (0.577), GNB (0.603), MLP (0.601), and CNB (0.543), with the XGBoost model having the largest AUC, the model can be applied in clinical practice.

**Conclusion:**

In this research, all five ML models were found to predict the risk of nodular thyroid disease in coal miners, with the XGBoost model having the best overall predictive performance. The model can assist clinicians in quickly and accurately predicting the occurrence of nodular thyroid disease in coal miners, and in adopting individualized clinical prevention and treatment strategies.

## Introduction

Coal is one of the world’s most important energy resources and coal mining and processing is a dust-producing process, and coal power is still the main electricity supply structure in China (1, 2). Coal dust is one of the main sources of health hazards for coal miners (3). Ramis et al. (4) found that mine workers with exposure to underground coal were significantly more likely to develop thyroid cancer than workers in other work environments. Nodular thyroid disease is strongly associated with an increased risk of thyroid cancer, and nodular thyroid disease has become one of the leading causes of death and disease burden in people worldwide due to thyroid cancer (5–8). Coal miners who are exposed to coal dust in long-term underground operations are at high risk of developing nodular thyroid disease. Early and effective recognition of risk factors for nodular thyroid disease and effective screening is an important step in improving the occupational health of coal miners.

Currently, there is a lack of extensive diagnostic resources for nodular thyroid disease, which is mainly detected by thyroid ultrasound. However, the shortage of thyroid ultrasonographers and the regional imbalance in the level of medical technology are the main current issues in the implementation of mass screening with thyroid ultrasound. The application of Artificial Intelligence (AI) machine learning technology to the screening of coal miners for nodular thyroid disease could therefore help to alleviate the current problem of thyroid ultrasound. The aim of this study is to develop and validate a simple and high performance artificial intelligence machine learning model to assist clinicians in early, rapid and accurate prediction of the risk of developing nodular thyroid disease in coal miners exposed to high risk environments for long-term exposure to the disease, leading to a personalized clinical prediction strategy.

Machine learning is a component of AI and is described as the process by which a computer learns from experience and performs a predetermined task without prior knowledge (9). Machine learning as a new AI technique has been widely used in the diagnosis and prediction of diseases (10–12). In some cases, the robustness and predictive power of ML algorithms outperform traditional statistical modeling, so it may be possible to predict the risk of nodular thyroid disease in coal miners more efficiently using ML models. For the time being, no researcher has used machine learning models to predict the risk of developing nodular thyroid disease in coal miners. In a previous related study, Zhou et al. (13) used machine learning to predict the malignancy of nodular thyroid disease based on ultrasound images, but the clinical application of this research still needs to rely on ultrasound images for prediction, while at the same time, the machine learning model of this study is universally applicable. The ML models constructed in this study based on clinical and imaging data of coal miners are specific to coal miners who are exposed to high risks for a long period of time, and the final ML models were selected with high accuracy and predictive power.

This study compared the performance of five machine learning models, Extreme Gradient Augmentation (XGBoost), Logistic Classification (LR), Gaussian Parsimonious Bayesian Classification (GNB), Neural Network Classification (MLP), and Complementary Parsimonious Bayesian Classification (CNB) models, in predicting different parameters for the occurrence of nodular thyroid disease in coal miners, and selected the best ML models among them, which can be applied in clinical practice activities in the next step and guide clinicians to take more targeted predictive treatment measures.

## Materials and methods

### Ethical approval

This clinical study was a retrospective clinical study, conducted in accordance with the ethical standards of the World Medical Association Declaration of Helsinki, and all personally identifiable information was encrypted by the investigators so as not to disclose personal privacy. The study was applied for and ethically approved by the ethical review committee of the First People’s Hospital of Anhui University of Technology (Huainan First People’s Hospital) (approval number: 2022-YJ-020-01), and all study subjects had an exemption from informed consent.

### Study population

This study collected clinical examination data from 1,708 coal miners from 31 different coal mining companies in Huainan, Anhui Province, China, who were examined in April 2021 at the Occupational Disease Prevention and Control Institute in Huainan, Anhui Province, China. All coal miners were retrospectively analyzed for clinical baseline characteristics and factors influencing the occurrence of nodular thyroid disease.

### Inclusion and exclusion criteria

Entry criteria: (1) Age ≥18 years old. (2) Male coal miners with ≥3 years of service. (3) No previous serious organic lesions. (4) In addition to the usual physical examination items, the physical examination should also include color Doppler ultrasound examination of the thyroid gland. (5) The medical examination is complete and free of missing information. Exclusion criteria: (1) Persons with serious cardiovascular, cerebrovascular, hepatic, renal or other serious primary malignant diseases. (2) Those with severe mental disorders or other reasons that prevent them from cooperating with the medical examination. (3) Those who have a history of surgery or radiotherapy or chemotherapy for malignant tumors. (4) Those with serious infectious diseases of the systemic system. (5) Those without diagnostic thyroid ultrasound results or with incomplete information on the physical examination. Finally, 1,708 coal miners who met the study requirements and were included in the observation had their physical examination data collected, including general clinical information, laboratory test indicators and imaging findings.

### Diagnostic criteria

The diagnosis of nodular thyroid disease in this study was made by two or more ultrasonographers with more than 10 years of diagnostic experience, and was based on the Thyroid Imaging Reporting and Data System (TI-RADS) classification developed by the American College of Radiology (14, 15). The nodules are scored on the basis of their composition, internal echogenicity, morphology and margins12: (1) 0 points for no internal echogenicity, 1 point for isoechoic or strong echogenicity, 2 points for hypoechogenicity and 3 points for very low echogenicity; (2) 0 points for cystic or predominantly cystic echogenicity, 1 point for mixed echogenicity and 2 points for solidity; (3) 0 points for non-erect growth of nodules, 3 points for (3) 0 points for a non-erect nodule, 3 points for an upright nodule; (4) 0 points for a nodule with smooth margins, 2 points for an irregular or lobulated margin, and 3 points for a nodule invading the outer thyroid gland; (5) 0 points for no focal strong echogenicity or a “large comet tail” sign, 1 point for a nodule with a coarse calcified foci, 2 points for a peripheral calcified foci, and 3 points for a punctate strong echogenicity. 3 points. The nodule scores were summed to determine the classification criteria: 0 for TI-RADS category 1, with a probability of malignancy of ≤2%; 2 for TI-RADS category 2, with a probability of malignancy of ≤2%; 3 for TI-RADS category 3, with a probability of malignancy of ≤5%; 4–6 for TI-RADS category 4, with a probability of malignancy of 5–20%; and ≥7 for TI-RADS category 5, with a probability of malignancy of ≥20%.

### Data collection

General information on coal miners includes age and gender. Laboratory indicators included liver function indicators (total protein, glutathione, albumin, globulin, albumin globule ratio, total bilirubin, direct bilirubin, indirect bilirubin, ghrelin, ghrelin/glutathione ratio, alkaline phosphatase, and glutamyl transferase); renal function indicators (creatinine, urea, and blood uric acid); lipid indicators (LDL cholesterol, triglycerides, HDL cholesterol, and total cholesterol), tumor markers (alpha-fetoprotein, carcinoembryonic antigen, total prostate-specific antigen, and cytokeratin 19 fragment); white blood cell count and its classification count (leucocytes, monocytes, basophils, eosinophils, absolute values of immature granulocytes, and neutrophils); red blood cell count (red blood cells, red blood cell distribution width CV, red blood cell distribution width SD, Erythrocyte pressure, mean erythrocyte volume, mean erythrocyte hemoglobin volume, and mean erythrocyte hemoglobin concentration); hemoglobin count indicators (hemoglobin, mean hemoglobin content, and mean hemoglobin concentration); platelet count indicators (platelets, large platelet ratio, platelet distribution width, mean platelet volume, and platelet pressure); other indicators (myeloperoxidase, lipoprotein related phospholipase A2, fasting blood glucose, and Antibodies to *Helicobacter pylori*).

### Data analysis

R software version 3.6.3 and python software version 3.7 were used for statistical analysis of the data and machine learning classification modeling. Count data were expressed as frequencies and percentages (%), and the χ^2^ test was used for comparison between groups; measurement data conforming to normal distribution were expressed as (−x ± s), and measurement data not conforming to normal distribution were expressed as M (P_25_, P_75_), and the Mann–Whitney U test was used for comparison between groups. *p* < 0.05 was considered a statistically significant difference.

**FIGURE 1:**
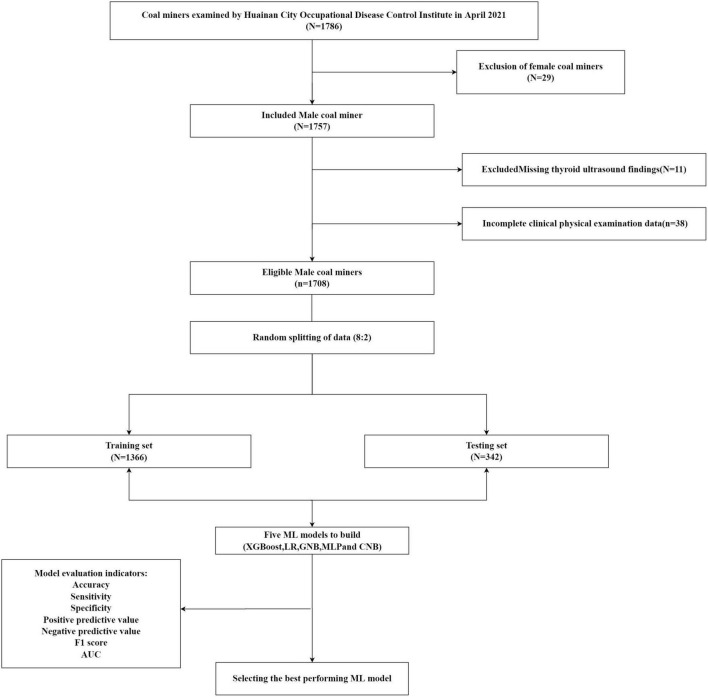
Enrollment of coal miners and data processing flow chart.

In this study, we randomly divided the dataset into two groups, the Training dataset for ML model development (80%) and the Test dataset for performance evaluation (20%). A five times resampling method was used for the analysis of different ML models. Five different ML models were used to analyze the data: XGBoost, LR, GNB, MLP, and CNB models. During the ML model training process, to better compare multiple models, we used a 5-fold resampling method the consistency of the training samples when different models were trained. The prediction performance of the five different ML models was assessed by comparing the accuracy, sensitivity, specificity, positive predictive value, negative predictive value, F1 score and area under the receiver operating characteristic curve (AUC) of each ML classification model in the Test dataset. The ML data analysis in this study was based on python “xgboost 1.2.1,” “lightgbm 3.2.1,” “sklearn 0.22.1,” “imblearn,” and the R “logreg 6.2.0,” “statsmodels 0.11.1,” packages are complete. The flow of data collection and work processing for coal miners is shown in Figure 1.

## Results

### Baseline features

A total of 1,708 eligible coal miners with a mean age of 41.39 ± 8.28 years (18–60 years) were included in this study. Of these, 578 cases (33.84%) were in the nodular thyroid disease group and 1,130 cases (66.16%) were in the no nodular thyroid disease group. The data were balanced using the synthetic minority oversampling technique (SMOTE) method, resulting in a positive to negative sample ratio of 2:1. The final matched results were 1130 cases in the nodular thyroid disease group and 2260 cases in the no-nodular thyroid disease group. The differences in AGE, RBC, RDW-SD, MCV, MCH, and GGT between the two groups of nodular thyroid disease were statistically significant at *p* < 0.05 (Table 1).

**TABLE 1:** Baseline clinical characteristics of the two groups of coal miners.

Variables	Non-nodular thyroid disease (*n* = 1,130)	Nodular thyroid disease (*n* = 578)	Total data (*n* = 1,708)	Statistic	*P*-value
Age, year, M (P_25_,P_75_)	40 (34,48)	43 (36,50)	41 (34,49)	–4.739	<0.001
LDL-C, mmol/L, M (P_25_,P_75_)	2.8 (2.39,3.28)	2.83 (2.4,3.32)	2.81 (2.39,3.29)	–0.740	0.459
TG, mmol/L, M (P_25_,P_75_)	1.32 (0.9,2.03)	1.36 (0.94,2.17)	1.33 (0.91,2.07)	–1.595	0.111
HDL-C, mmol/L,M (P_25_,P_75_)	1.16 (1.02,1.34)	1.16 (1.02,1.35)	1.16 (1.02,1.34)	–0.101	0.919
TC, mmol/L, M (P_25_,P_75_)	4.92 (4.38,5.58)	5.01 (4.37,5.61)	4.95 (4.37,5.6)	–1.069	0.285
Cr, mmol/L, M (P_25_,P_75_)	69.9 (63.7,76.3)	71 (64.4,76)	70.2 (63.9,76.1)	–1.276	0.202
BU, mmol/L, M (P_25_,P_75_)	5.26 (4.57,6.2)	5.18 (4.45,6.1)	5.22 (4.5,6.16)	1.553	0.121
AFP, μg/L, M (P_25_,P_75_)	3.46 (2.24,4.57)	3.59 (2.45,4.83)	3.5 (2.3,4.69)	–1.881	0.06
CEA, μg/L, M (P_25_,P_75_)	1.95 (1.43,2.64)	1.98 (1.41,2.68)	1.96 (1.43,2.65)	–0.071	0.943
WBC, 10^9^/L, M (P_25_,P_75_)	6.47 (5.62,7.59)	6.52 (5.67,7.74)	6.5 (5.62,7.64)	–1.052	0.293
P-LCR, %, M (P_25_,P_75_)	32.53 (32.19,32.96)	32.5 (32.17,32.93)	32.52 (32.19,32.95)	0.335	0.737
MONO, %, M (P_25_,P_75_)	0.39 (0.33,0.47)	0.39 (0.32,0.48)	0.39 (0.32,0.48)	–0.518	0.604
RBC,10^12^/L, M (P_25_,P_75_)	4.96 (4.74,5.21)	4.91 (4.7,5.17)	4.95 (4.72,5.2)	2.395	0.017
RDW-CV, %, M (P_25_,P_75_)	12.6 (12.3,12.9)	12.6 (12.3,12.9)	12.6 (12.3,12.9)	–0.163	0.871
RDW-SD, fL, M (P_25_,P_75_)	43.6 (42.32,44.9)	43.9 (42.5,45.3)	43.7 (42.4,45)	–2.804	0.005
HCT, %, M (P_25_,P_75_)	45.9 (44.1,47.6)	45.7 (44,47.4)	45.8 (44,47.6)	1.017	0.309
LYMPH, 10^9^/L, M (P_25_,P_75_)	2.14 (1.78,2.6)	2.16 (1.78,2.64)	2.15 (1.78,2.61)	–0.563	0.574
MCV, fL, M (P_25_,P_75_)	92.3 (90.1,94.8)	92.7 (90.5,95.3)	92.5 (90.2,95)	–2.321	0.02
MCH, pg, M (P_25_,P_75_)	31.6 (30.72,32.5)	31.8 (30.9,32.7)	31.6 (30.8,32.55)	–2.724	0.006
MCHC, g/L, M (P_25_,P_75_)	342 (339,345)	342 (339,346)	342 (339,345)	–1.927	0.054
BASO, 10^9^/L, M (P_25_,P_75_)	0.03 (0.02,0.04)	0.03 (0.02,0.04)	0.03 (0.02,0.04)	0.780	0.436
EOS, 10^9^/L, M (P_25_,P_75_)	0.13 (0.08,0.21)	0.14 (0.08,0.23)	0.13 (0.08,0.22)	–0.577	0.564
IG, 10^9^/L, M (P_25_,P_75_)	0.02 (0.01,0.03)	0.02 (0.01,0.04)	0.02 (0.01,0.03)	–1.509	0.131
HGB, g/L, M (P_25_,P_75_)	157 (150,163)	157 (150,163)	157 (150,163)	0.549	0.583
PLT, 10^9^/L, M (P_25_,P_75_)	218 (188,252)	220 (188,252)	219 (188,252)	–0.548	0.583
PDW, fL, M (P_25_,P_75_)	16.2 (16,16.4)	16.2 (16,16.4)	16.2 (16,16.4)	1.018	0.309
MPV, fL, M (P_25_,P_75_)	10.4 (9.6,11.2)	10.2 (9.6,11.1)	10.3 (9.6,11.2)	1.649	0.099
PCT, %, M (P_25_,P_75_)	0.22 (0.2,0.26)	0.23 (0.2,0.26)	0.22 (0.2,0.26)	–0.086	0.932
NEUT, 10^9^/L, M (P_25_,P_75_)	3.67 (3.03,4.44)	3.68 (3.03,4.55)	3.67 (3.03,4.48)	–0.728	0.467
TP, g/L, M (P_25_,P_75_)	32.6 (6.9,71.28)	40.15 (7.3,73.55)	34.6 (7.07,72.15)	–0.960	0.337
PSA, ng/mL, M (P_25_,P_75_)	0.76 (0.54,1.09)	0.76 (0.53,1.03)	0.76 (0.54,1.07)	0.787	0.432
AST, U/L, M (P_25_,P_75_)	21.5 (18.5,25.9)	22 (18.6,26)	21.7 (18.5,25.9)	–1.043	0.297
ALB, g/L, M (P_25_,P_75_)	46.75 (45.28,48.26)	46.69 (44.9,48.09)	46.71 (45.15,48.2)	1.616	0.106
A/G, M (P_25_,P_75_)	1.61 (1.46,1.75)	1.6 (1.43,1.73)	1.6 (1.45,1.74)	1.588	0.112
GGT, U/L, M (P_25_,P_75_)	28.45 (20.7,44.73)	30.8 (21.7,48.7)	28.9 (20.9,45.8)	–2.254	0.024
ALT, U/L, M (P_25_,P_75_)	21.45 (15.47,30.8)	21.6 (15.3,30.8)	21.5 (15.4,30.8)	–0.414	0.679
AST/ALT, M (P_25_,P_75_)	1.00 (0.78,1.29)	0.99 (0.77,1.31)	1.00 (0.78,1.3)	0.254	0.799
IBIL,umol/L,M (P_25_,P_75_)	9.8 (7.4,12.8)	9.8 (8.1,12.9)	9.8 (7.6,12.8)	–1.144	0.253
ALP, U/L, M (P_25_,P_75_)	73.7 (62.6,86.82)	74.6 (63.5,85.6)	74 (62.9,86.2)	–0.850	0.395
Glo,g/L,M (P_25_,P_75_)	29.01 (27.07,31.46)	29.48 (27.1,31.81)	29.11 (27.1,31.56)	–1.341	0.18
DBil, μmol/L, M (P_25_,P_75_)	3.9 (2.9,5.1)	3.9 (3.1,5.2)	3.9 (3,5.1)	–0.678	0.498
TBil, μmol/L, M (P_25_,P_75_)	13.8 (10.5,17.9)	13.8 (11.3,17.9)	13.8 (10.7,17.9)	–1.076	0.282
TP, g/L, M (P_25_,P_75_)	75.7 (73.31,78.62)	75.89 (73.2,78.51)	75.8 (73.3,78.58)	–0.155	0.877
FBG, mmol/L, M (P_25_,P_75_)	5.99 (5.67,6.43)	6.01 (5.66,6.53)	5.99 (5.67,6.47)	–0.958	0.338
BUA, μmol/L, M (P25,P75)	328.65 (278.28,381.92)	327.3 (280.5,381.3)	328.4 (279.1,381.6)	–0.345	0.73
CYFRA21-1, ng/ml, M (P_25_,P_75_)	2.25 (2,2.57)	2.24 (2.01,2.57)	2.25 (2.01,2.57)	0.123	0.902
MPO, U/ml, M (P_25_,P_75_)	74.13 (58.98,99.54)	75.41 (60.48,98.89)	74.89 (59.2,99.41)	–0.767	0.443
Lp-PLA2, μg/L, M (P_25_,P_75_)	324.2 (241.74,416.88)	327.16 (248.7,406.25)	325.69 (243.64,413.9)	–0.382	0.703

LDL-C, low-density lipoprotein cholesterol; TG, triglycerides; HDL-C, high-density lipoprotein cholesterol; TC, total cholesterol; Cr, creatinine; BU, blood urea; AFP, alpha-fetoprotein; CEA, carcinoembryonic antigen; WBC, leukocytes; P-LCR, large platelet ratio; MONO, mononuclear cells; RBC, red blood cells; RDW-CV, red blood cell distribution width CV; RDW-SD, red blood cell distribution width; HCT, erythrocyte pressure volume; LYMPH, lymphocytes; MCV, mean red blood cell volume; MCH, average red blood cell hemoglobin volume; MCHC, mean red blood cell hemoglobin concentration; BASO, basophils; EOS, eosinophils; IG, absolute value of immature granulocytes; HGB, hemoglobin; PLT, blood platelets; PDW, platelet distribution width; MPV, mean platelet volume; PCT, platelet pressure; NEUT, neutrophils; TP, total protein; PSA, total prostate-specific antigen; AST, glutathione transaminase; ALB, serum albumin; A/G, albumin/globulin; GGT, glutamyl transpeptidase; ALT, glutathione aminotransferase; AST/ALT, glutathione/glutathione transaminase; IBIL, indirect bilirubin; ALP, alkaline phosphatase; Glo, globulin; DBil, direct bilirubin; TBil, total bilirubin; TP, total protein; FBG, plasma fibrinogen; BUA, blood uric acid; CYFRA21-1, cytokeratin 19 fragment; MPO, myeloperoxidase; Lp-PLA2, lipoprotein-associated phospholipase A2.

Of the 578 miners with nodular thyroid disease, 296 (51.21%) were in TR-RADS category 2, 265 (45.85%) in TR-RADS category 3 and 17 (2.94%) in TR-RADS category 4. The analysis showed that age, MCH, MCV and RDW-SD were associated with the development of nodular thyroid disease in coal miners, all at *p* < 0.05, with statistically significant differences between groups (Table 2).

**TABLE 2:** Baseline characteristics of coal miners with nodular thyroid disease classification.

Variables	TR-RADS 1 (*n* = 1,130)	TR-RADS 2 (*n* = 296)	TR-RADS 3 (*n* = 265)	TR-RADS 4 (*n* = 17)	*P*-value
AGE, year, M (P25,P75)	40.00 (34.00,48.00)	41.00 (34.00,50.00)	46.00 (38.00,51.00)	42.00 (33.00,48.00)	<0.001***
ALP, U/L, M (P25,P75)	319.00 (156.00,491.00)	369.00 (189.00,490.00)	282.00 (112.00,463.00)	437.00 (225.00,477.00)	0.052
GGT, U/L, M (P25,P75)	326.00 (148.00,488.00)	292.00 (136.00,453.00)	378.00 (176.00,517.00)	306.00 (218.00,489.00)	0.028
PLT, 10^9^/L, M (P25,P75)	218.00 (188.00,252.00)	223.00 (192.00,261.00)	218.00 (188.00,243.00)	224.00 (188.00,233.00)	0.426
MCH, median (IQR)	31.60 (30.70,32.50)	31.80 (30.90,32.50)	31.80 (30.90,32.80)	32.10 (31.40,33.10)	0.024*
MCV, fL, M (P25,P75)	92.30 (90.10,94.80)	92.60 (90.40,95.00)	92.90 (90.50,95.50)	94.20 (90.90,95.60)	0.096
HCT, %, M (P25,P75)	45.90 (44.10,47.60)	45.60 (43.70,47.20)	45.80 (44.10,47.80)	46.50 (45.00,47.30)	0.128
RDW-SD, fL, M (P25,P75)	43.60 (42.30,44.90)	43.90 (42.50,45.20)	44.00 (42.40,45.40)	44.70 (42.90,45.10)	0.045*
RDW-CV,%, M (P25,P75)	12.60 (12.30,12.90)	12.60 (12.40,12.90)	12.60 (12.30,12.90)	12.60 (12.40,12.80)	0.911
RBC, 1,012/L, M (P25,P75)	4.96 (4.74,5.21)	4.90 (4.69,5.15)	4.92 (4.71,5.18)	4.92 (4.76,5.21)	0.071
HDL-C, mmol/L, M (P25,P75)	69.00 (25.00,103.00)	69.00 (28.00,105.00)	62.00 (26.00,99.00)	72.00 (51.00,99.00)	0.544
TG, mmol/L, M (P25,P75)	198.00 (115.00,294.00)	194.00 (103.00,283.00)	210.00 (121.00,295.00)	117.00 (73.00,273.00)	0.354
LDL-C, mmol/L, M (P25,P75)	152.00 (66.00,237.00)	140.00 (72.00,219.00)	173.00 (78.00,249.00)	140.00 (74.00,243.00)	0.203

*p < 0.05, ***p < 0.001.

### Lasso regression screening of machine learning model predictor variables

**FIGURE 2:**
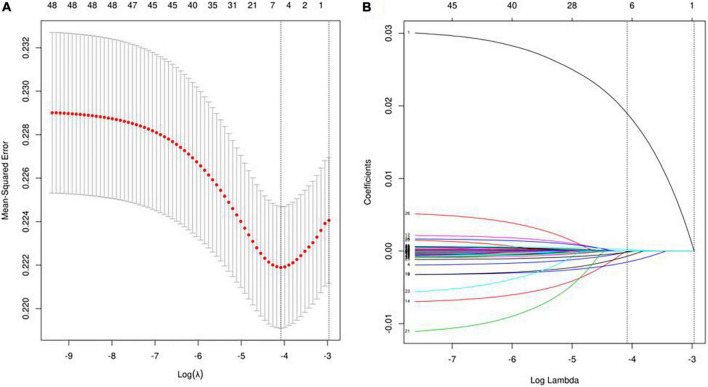
Lasso regression screening of ML model predictor variables. Panel **(A)** shows the process of screening for the most appropriate λ in the lasso model; panel **(B)** shows the lasso coefficient curves for the 48 variables.

Lasso regression was used to screen the predictor variables for the ML model, and the cross-validated Lasso fit Mean-Squared Error (MSE) plot (Figure 2A) and the Lasso fit coefficient trajectory plot (Figure 2B) were used. The value corresponding to when the loss function achieves a minimum is determined to be the optimal λ value based on the lowest point in Figure 2A, and the variables intersecting the optimal λ value are combined with Figure 2B to yield the final inclusion in the model variables. Therefore, when the minimum mean square error of λ is 0.017, the corresponding model predictor variables are selected as Age, HDLC, HCT, MCH, PLT, and GGT.

**FIGURE 3:**
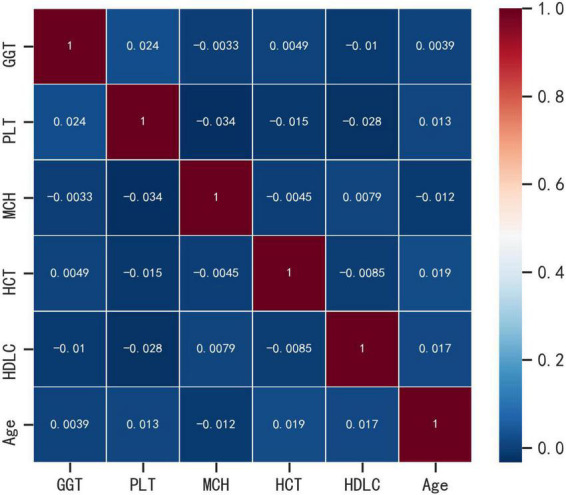
Correlation heat map analysis.

**FIGURE 4:**
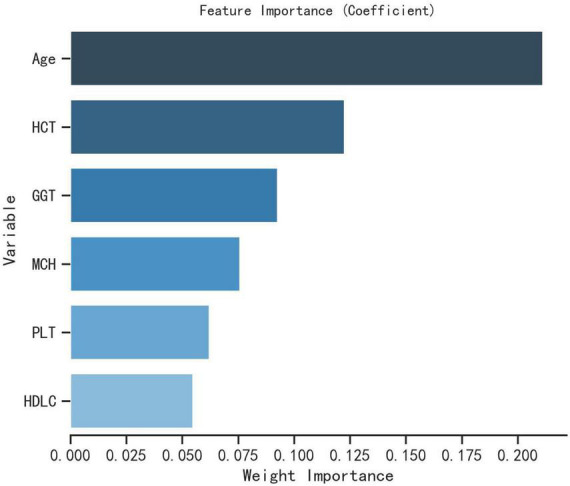
Importance analysis of XGBoost model variables.

Spearman correlation was used to test the relationship between the above variables and the correlation heat map showed no significant correlation between the variables, indicating that the variables were independent of each other (Figure 3). The importance of each variable in the ML algorithm varied, and we used an extreme gradient boosting tree (XGBOOST) to conduct a variable importance analysis of all the variables, screening out the variables with high correlation and ranking in the top six, and the results showed that the six variables with the highest importance (from highest to lowest) were Age, HCT, GGT, MCH, PLT, and HDL-C (Figure 4). Interestingly, the top six most important variables screened in the XGBoost model were consistent with the predictor variables screened by Lasso regression, and these variables will be used in the next step for machine learning classification modeling.

### Comparison of the predictive performance of five machine learning models

The evaluation metrics: accuracy, sensitivity, specificity, positive predictive value, negative predictive value, F1 score, and AUC (Table 3) of the five machine learning models XGBoost, LR, GNB, MLP, and CNB were compared in the training and Test dataset to evaluate the model prediction performance for the occurrence of nodular thyroid disease in coal miners. The results showed that the highest F1 value was for the XGBoost model, followed in order by the LR model, GNB model, MLP model and the smallest for the CNB model.

**TABLE 3:** Efficacy results for the five ML models.

Evaluation indicators	Training dataset (80%)					Test dataset (20%)				
	LR	GNB	CNB	MLP	XGBoost	LR	GNB	CNB	MLP	XGBoost
Accuracy (%)	65.0	63.3	53.9	61.6	91.6	62.5	61.8	55.7	61.3	81.8
Sensitivity (%)	79.4	68.1	55.3	67.7	90.2	70.7	66.2	56.9	65.6	75.1
Specificity (%)	36.6	53.7	51.2	49.5	94.5	46.0	53.4	53.2	53.1	96.4
Positive predictive value (%)	71.3	74.6	69.5	72.9	97.0	73.1	74.2	70.4	74.2	97.8
Negative predictive value (%)	47.3	45.9	36.3	44.3	83.1	43.6	43.7	39.0	44.0	64.4
F1 score	0.75	0.71	0.62	0.70	0.94	0.72	0.70	0.62	0.69	0.85
AUC(95%CI)	0.58 (0.56–0.60)	0.62 (0.60–0.65)	0.53 (0.51–0.55)	0.61 (0.58–0.63)	0.98 (0.97–0.98)	0.58 (0.53–0.63)	0.60 (0.56–0.65)	0.54 (0.50–0.59)	0.60 (0.56–0.65)	0.89 (0.87–0.92)

LR, Logistics Classification; GNB, Gaussian Parsimonious Bayesian Classification; CNB, Complementary Parsimonious Bayesian Classification; MLP, Neural Network Classification; XGBoost, Extreme Gradient Boosting Tree; AUC, area under the ROC curve.

**FIGURE 5:**
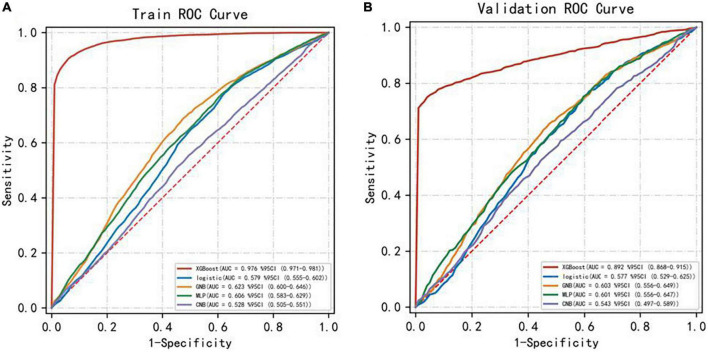
AUC for the five ML models. Panel **(A)** represents the training dataset ROC curve and panel **(B)** represents the test dataset ROC curve.

In comparing the performance of ML models, the closer the AUC is to 1, the better the performance of the classification model. The AUC of the five machine learning models in the Test dataset are shown in Figure 5, and the comparison results show that: XGBoost model > GNB model > MLP model > LR model > CNB model. The AUC of XGBoost model > 0.75 indicates that the model has good prediction performance. Both evaluation methods show that the XGBoost model has the best prediction performance and the CNB model has the worst prediction performance.

## Discussion

Nodular thyroid disease has become one of the most common thyroid disorders and is very prevalent worldwide (16, 17). Environmental factors are determinants of the development of nodular thyroid disease in people. In order to reduce the risk of nodular thyroid disease in people in specific environments, it is necessary to understand the relationship between environmental factors and thyroid dysfunction (18). Studies (19, 20) have found that environmental factors can activate the innate immune response leading to the development of thyroid disease. Not only that, but thyroid nodules (TNS) are one of the very common thyroid disorders, with nodular thyroid disease reported to be found in 3–7% of adults worldwide (21, 22). Currently, thyroid ultrasound is the test of choice for the diagnosis of nodular thyroid disease (23). Thyroid ultrasound is used to assess the malignancy grade of thyroid nodules by scoring all indicators of thyroid nodules together and finally according to the overall score. The diagnosis of nodular thyroid disease, the dependent variable indicator in this study, is clearly diagnosed by thyroid ultrasound. However, in the real world, nodular thyroid disease is often overlooked because it may occur early without any clinical symptoms. The underground environment in which coal miners work is complex, and many complex environmental factors (such as enclosed dust, ionizing radiation, exposure to harmful heavy metals, etc.) can cause disturbances in the body metabolism of miners, leading to the development of nodular thyroid disease. However, there are no studies by scholars that have used machine learning models to predict the occurrence of nodular thyroid disease in coal miners.

We screened six easily available parameters, Age, HCT, GGT, MCH, PLT, and HDLC, based on physical and clinical data of coal miners from 31 different coal mining companies in Huainan, Anhui Province, China, and applied these parameters to five different machine learning XGBoost, LR, GNB, MLP, and CNB models. This study developed and validated an ML algorithm model that can accurately predict the occurrence of thyroid disease in coal miners, which can provide effective prediction for accurate clinical treatment. The AUCs of all five models were greater than 0.5, indicating that coal miners with underground operations have a higher risk of developing nodular thyroid disease than the general population, with the XGBoost model being the optimal prediction model.

Logistics classification is a machine learning classification algorithm used to determine the risk probability of a disease and its results can be binomial or polynomial, LR is used for the creation of statistical models of binary data (24). In medicine, LR is often the most commonly used method for binomial outcome prediction models (25). Bayesian is an emerging technique in medical research, and it is widely used in public health research (26). Bayesian is a machine learning method for inductive inference and Bayesian algorithms play a crucial role in data observation studies, where the complementary plain Bayesian CNB allows a more even use of the amount of training data per category and more objectivity in the estimation results, which can reduce bias in the estimation (26–28). We obtained more stable weight estimates and higher classification accuracy. In contrast, MLP is a machine learning computational model inspired by biological neural networks, which is most commonly used in artificial intelligence models for risk prediction and assessment (25, 29). GBoost is a scalable tree-advancing machine learning algorithm based on decision trees (30). The XGBoost model is now widely used for regression and classification in a variety of data mining fields, and is widely used by data scientists to solve many advanced problems due to its high flexibility and excellent performance as a state-of-the-art gradient augmentation (GB) system (30, 31). Tomoo et al. (32) compared three machine learning algorithms, XGBoost, logistic regression and decision trees, to predict neurological recovery in patients with cervical spinal cord injury, and found that XGBoost achieved satisfactory accuracy compared to the other two traditional machine learning models, which had good predictions on the ROC curve.

Compared to the other four machine learning models, the XGBoost model has the following advantages: it has no explicit linearity requirement for the data distribution and automatically detects non-linear relationships and interaction effects using the relevant factors; it can make full use of missing data without filling in the data and can more realistically reflect the original results expressed by the data; the XGBoost model has the advantage of handling large samples of data (33), learning and remembering as it analyses and processes the data and improving its predictive power; the model trained with a large amount of data is more reliable than a machine learning model fitted to a small sample of individual tests (34). The XGBoost model has the advantage of being able to learn and remember as the data is analyzed and processed, and to improve its predictive power (35); a model trained on a large amount of data is more reliable than a machine learning model fitted to a small sample of individual tests, and has a high clinical application in predicting classification outcomes (36). In this study, five machine learning models were attempted for the prediction of nodular thyroid disease in coal miners, and the results showed that the XGBoost model achieved good prediction results.

This study evaluated the predictive performance of different models by combining various metrics such as accuracy, sensitivity, specificity, positive predictive value, negative predictive value, F1 score, and AUC, which to a certain extent reduces the study bias caused by a single model or a single evaluation metric. the ROC curve is used to summarize the performance of a model by evaluating the value between the false positive rate (1-specificity) and the true positive rate (sensitivity). The AUC is a measure of the performance of the ROC curve; the higher the AUC, the better the predictive ability of the model. The results of the study showed that in the Test dataset, the five machine learning models had accuracy (55.7–81.8%), sensitivity (56.9–75.1%), specificity (46.0–96.4%), positive predictive value (70.4–97.8%), negative predictive value (39.0–64.4%), F1 score (0.62–0.85) and AUC values (0.54–0.89). Among them, the XGBoost model had 81.8% accuracy, 75.1% sensitivity, 96.4% specificity, 97.8% positive predictive value, 64.4% negative predictive value, F1 score of 0.8549 and AUC value of 0.89. On comprehensive analysis, it can be found that the indicators of the XGBoost model were significantly greater than those of the other four ML models, indicating that the XGboost model showed the strongest predictive effect in the Test dataset, further inferring that the XGboost model had the strongest overall strength and was the best model for predicting the risk of nodular thyroid disease in coal miners.

In the research, five different machine learning models were developed and validated to predict the occurrence of nodular thyroid disease in coal miners based on their clinical and imaging indicators, and the best ML model was selected. The clinical application of this ML model can, to a certain extent, alleviate the shortage of ultrasonographers and effectively reduce the misdiagnosis or underdiagnosis of nodular thyroid disease due to human factors, thus greatly improving the efficiency and accuracy of clinical diagnosis.

However, there are some limitations that need to be taken into account when interpreting the results of this study. Firstly, the data collection of coal miners lacked behavioral data on the living and working conditions of coal miners and some blood biochemical indicators such as history of alcohol consumption, smoking, family history, body mass index, length of service, number of night shift days, thyroid hormones, blood sedimentation, and other relevant data, which may lead to some confounding effects. Second, the fixed nature of the items in each coal mining company’s medical examination package prevented us from obtaining histopathological examinations of thyroid disease, and this study also lacked image-based machine learning data, resulting in a less comprehensive analysis. Third, this study used the synthetic minority class oversampling technique (SMOTE) to balance the proportion of positive and negative samples. The basic idea of SMOTE is to analyze minority class samples and manually synthesize new samples to add to the dataset based on minority class samples, so the analyzed model results may be prone to overfitting, and the next step in our study will be to further increase the positive and negative sample size to bring the model closer to the true The next step of our study will be to further increase the positive and negative sample sizes to bring the model closer to the true predictions. Fourth, although the main aim of this study was to explore the feasibility of different machine learning models to predict thyroid disease in coal miners, other machine learning models, such as random forest, support vector machine and decision tree, can be added in future studies to further explore and compare the performance of different machine learning models and advance the application of machine learning in predicting the occurrence of thyroid disease in coal miners. Fifth, most of the coal miner data in this study came from Anhui Province, China, and coal miner data from other geographical regions in China were not included, and the model results may be subject to geographical bias. In addition, this study did not use an external dataset for model validation; all validations used a 5-fold resampling technique to split this dataset into a test and Test dataset at a ratio of 8:2. The inclusion of an external Test dataset could be an avenue for future research.

## Summary and conclusion

In this study, for the first time, different machine learning models were used to predict the risk of nodular thyroid disease in coal miners, and among the five ML models, the XGBoost model had the highest predictive performance for nodular thyroid disease in coal miners. This ML model can be used by clinicians to assess the high risk of nodular thyroid disease in coal miners at an early stage in the practice of medicine, and to adopt targeted clinical treatment strategies.

## Data availability statement

The original contributions presented in this study are included in the article/supplementary material, further inquiries can be directed to the corresponding author.

## Ethics statement

This clinical study was a retrospective clinical study, conducted in accordance with the Ethical Standards of the World Medical Association Declaration of Helsinki, and all personally identifiable information was encrypted by the investigators so as not to disclose personal privacy. The study was applied for and ethically approved by the Ethical Review Committee of The First People’s Hospital of Anhui University of Technology (Huainan First People’s Hospital) (approval number: 2022-YJ-020-01), and all study subjects had an exemption from informed consent.

## Author contributions

DC and WW contributed to the conception and design of this study. FZ contributed to the writing of the manuscript. HZ designed the study and statistical analysis. YL, YW, DL, and CD contributed to data retrieval and manuscript review. DR and LY contributed to data collection and data collation. All authors made significant contributions to the research process of this manuscript and have read and approved the submitted manuscript.
